# Aescin Protects against Experimental Benign Prostatic Hyperplasia and Preserves Prostate Histomorphology in Rats via Suppression of Inflammatory Cytokines and COX-2

**DOI:** 10.3390/ph15020130

**Published:** 2022-01-22

**Authors:** Mohamed Raafat, Amr A. Kamel, Alaa H. Shehata, Al-Shaimaa F. Ahmed, Asmaa M. A. Bayoumi, Rabab A. Moussa, Mohammed A. S. Abourehab, Mahmoud El-Daly

**Affiliations:** 1 Department of Pharmacology and Toxicology, Umm Al-Qura University, Makkah 21955, Saudi Arabia; 2 Department of Pharmacology and Toxicology, Faculty of Pharmacy, Minia University, El-Minia 61519, Egypt; amousa6@uwo.ca (A.A.K.); alaa_fawzy2344@yahoo.com (A.H.S.); shaimaa.faissal@minia.edu.eg (A.-S.F.A.); 3 Department of Biochemistry, Faculty of Pharmacy, Minia University, El-Minia 61519, Egypt; asmaa_bayoumi@mu.edu.eg; 4 Department of Pathology, Faculty of Medicine, Minia University, El-Minia 61519, Egypt; rababmossa00@gmail.com; 5 Department of Pharmaceutics, College of Pharmacy, Umm Al-Qura University, Makkah 21955, Saudi Arabia; maabourehab@uqu.edu.sa

**Keywords:** aescin, glucocorticoid-like activity, benign prostatic hyperplasia, inflammation, IL-1β, TNF-α, COX-2, PCNA, TGF-β, testosterone

## Abstract

Background: Benign prostatic hyperplasia (BPH) is the most common urogenital condition in aging males, while inflammation and tissue proliferation constitute the main pathophysiological factors. The adverse effects of currently available BPH medications limit patient compliance. We tested the protective effect of aescin against the development of BPH in rats. Methods: A total of 18 male Wistar rats were divided into 3 groups: control (sesame oil 1 mL/kg, s.c.); BPH (testosterone oenanthate 3 mg/kg, s.c., in sesame oil), and BPH-aescin rats (testosterone oenanthate 3 mg/kg, s.c. + aescin 10 mg/kg/day, p.o.). All treatments continued for 4 weeks. Serum and prostatic samples were harvested for biochemical and histopathological examination. Results: Induction of BPH by testosterone increased the prostate weight and prostate weight index, serum testosterone, prostate expression of inflammatory (IL-1β, TNF-α, and COX-2), and proliferative markers (PCNA and TGF-β1). Concurrent treatment with aescin decreased the testosterone-induced increase in prostatic IL-1β, TNF-α, and COX-2 expression by 47.9%, 71.2%, and 64.4%, respectively. Moreover, aescin reduced the prostatic proliferation markers TGF-β1 and PCNA by 58.3% and 71.9%, respectively, and normalized the prostate weight. Conclusion: The results of this study showed, for the first time, that aescin protected against the development of experimental BPH in rats via its anti-inflammatory and antiproliferative effects. These findings warrant further studies to clinically repurpose aescin in the management of BPH.

## 1. Introduction

Benign prostatic hyperplasia (BPH) is a progressive inflammatory disorder characterized by abnormal prostate growth and urological symptoms [1]. BPH is common among males more than 50 years of age and affects approximately one in four males worldwide [2]. About 80% of lower urinary tract symptoms in males result from BPH-related pathology [2]. Enhanced growth of the transitional zone cells surrounding the urethra leads to prostate enlargement and bladder outlet obstruction, resulting in lower urinary tract symptoms (LUTS) [3]. Despite the advancement in the current medications for BPH treatment, finding new therapies is crucial. Currently, α_1_-adrenergic receptor antagonists and 5α-reductase inhibitors are widely used therapies in the treatment of BPH, and they may improve urological BPH-related symptoms [4,5]. Unfortunately, adverse effects, especially their effect on sexual performance, limit the clinical usefulness of such medications [5,6].

Clinical and experimental evidence implicates multiple overlapping mechanisms in the pathophysiology of BPH. These mechanisms involve the activation of inflammation and cellular proliferation. The common risk factors include aging, metabolic syndrome, systemic or inflammation, and altered hormone levels and/or sensitivity [1,7]. Together, these factors lead to inhibition of normal epithelial cell apoptosis, disrupted apoptotic and proliferative activities, and ultimately enhanced cellular proliferation, hyperplasia, and the incidence of prostatic cancer [1,7,8,9,10]. Careful targeting of such mechanisms should provide better therapeutic options, with fewer and less overwhelming adverse effects, and prevent the progression to prostate cancer.

Inflammation is one of the most important factors in the progression of BPH and prostate carcinoma [8,10,11,12,13,14]. Analysis of prostatic tissue samples from patients with BPH revealed numerous infiltrates of T and B lymphocytes and macrophages [8,15,16]. Both inflammation and prostatic enlargement strongly correlate with the extent and severity of BPH-associated urological symptoms [17,18]. The increased production of pro-inflammatory cytokines including tumor necrosis factor (TNF)-α and interleukins (e.g., IL1-β) during inflammation contributes to tissue remodeling in BPH, which ultimately induces epithelial and stromal cell hyperproliferation, activation of fibrosis-promoting signaling, and malignant transformation [7,12,13,14].

The increased oxygen demand of the proliferating prostate cells induces cellular hypoxia that may further the progression of BPH [19,20]. The hypoxic environment in prostatic stromal cells induces neovascularization and expression of multiple growth factors, particularly transforming growth factor (TGF)-β, vascular endothelial growth factor (VEGF), and fibroblast growth factor (FGF)-7 [1,9,19]. On the other hand, the increased expression of androgen receptors in human BPH cells augmented the infiltration of co-cultured monocytes/macrophages [21]. This co-culture system showed enhanced epithelial-to-mesenchymal (EMT) transition of epithelial cells downstream of increased TGF-β signaling. Previous work showed that infection-induced prostate inflammation increased the expression of androgen-responsive genes and TGF-β1 [22]. Besides, the age-related imbalance in the levels of sex hormones might potentiate the deleterious effects of TGF-β [23]. Regardless of its source, TGF-β is a potent suppressor of apoptosis that further interrupts the normal apoptotic process in favor of prostate tissue remodeling [9] and ultimately motivates carcinogenesis [1,24]. This effect manifests clinically as an increased proliferative index compared with a lowered apoptotic index of the prostate epithelium in BPH patients [25]. Others have found a positive correlation between the TGF-β type II receptor and prostate volume [26]. Taken together, TGF-β is critical in the pathogenesis of BPH and prostate cancer [24].

Aescin is a mixture of closely related triterpene saponins from *Aesculus hippocastanum* seeds. The therapeutically active isoform β-aescin exerts pleiotropic pharmacological effects [27,28,29]. It has potent anti-inflammatory, anti-edematous, antioxidant, and vasoprotective effects. The clinical uses of aescin include the management of hematoma and hemorrhoids [29]. The anti-inflammatory action of aescin involves modulation of the interaction between glucocorticoid receptor (GR) and inflammatory transcription factors, such as NFκB. Aescin upregulates GR expression and consequently suppresses the expression of inflammatory transcription factors-activated genes [30]. Additionally, the anti-proliferative effect of aescin is established in human hepatocellular carcinoma [31], osteosarcoma [32], and human oral carcinoma cells [33]. Aescin suppresses cell survival by activation of autophagy [32,34], DNA damage, and growth arrest at the G1/S phase [35]. Moreover, aescin potentiated the apoptotic and anti-proliferative effects of chemotherapeutic agents on cancer cells by inhibiting the expression and activity of various anti-apoptotic factors, such as Bcl-2, cyclin D1, survivin, and Bcl-xL [36]. Based on the reported anti-inflammatory and antiproliferative effects of aescin, we hypothesized that it would protect against the development of experimental BPH induced by testosterone administration.

## 2. Results

### 2.1. Effect of Aescin Treatment on Prostate Weight and Prostate Weight Index

**Figure 1:**
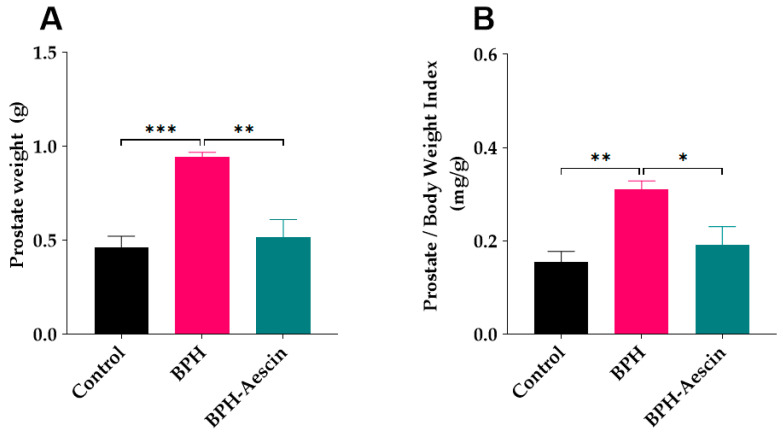
Aescin prevents testosterone-induced prostatic hyperplasia. The prostate glands were harvested from euthanized rats, dissected from surrounding tissues, and immediately weighed (g) on a sensitive balance (**A**). The prostate weight/body weight index (mg/g) was calculated as a ratio for each animal (**B**). Rats in control, BPH, and BPH–aescin groups received sesame oil (s.c.), testosterone (3 mg/kg, s.c.) dissolved in sesame oil, and testosterone (3 mg/kg, s.c.) dissolved in sesame oil + aescin (10 mg/kg, p.o.), respectively, for 4 weeks. Data (Mean ± SEM, *n* = 5) were analyzed by one-way analysis of variance (ANOVA) followed by Tukey’s multiple comparisons test. *, **, and *** denote significant differences between the selected study groups at *p* < 0.05, 0.01, and 0.001, respectively.

Data in Figure 1 show that induction of BPH by daily subcutaneous administration of testosterone significantly increased the prostate weight (0.94 ± 0.02 g, *p* < 0.001), and the prostate weight index (0.31 ± 0.01 mg/g, *p* < 0.01) compared with the control rats (0.46 ± 0.05 g and 0.15 ± 0.02 mg/g, respectively). Concomitant administration of aescin during the BPH induction period significantly decreased the prostate weight (0.51 ± 0.09 g, *p* < 0.01) and the prostate weight index (0.19 ± 0.03 mg/g, *p* < 0.05) compared with the untreated BPH rats.

### 2.2. Aescin Prevents the Testosterone-Induced Prostatic Histopathological Abnormalities

**Figure 2:**
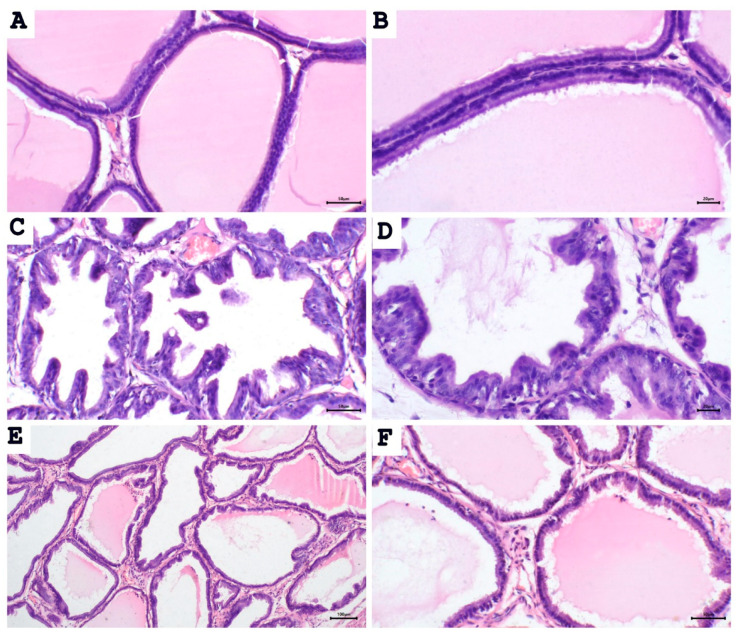
Aescin abrogates the testosterone-induced proliferation in the prostate. Sections of rat prostate stained with hematoxylin–eosin from the control, BPH, and BPH rats treated with aescin (10mg/kg, p.o.) for 4 weeks are shown. (**A**), (**B**): Prostatic sections from the control group show the normal histoarchitecture of the prostate gland with regular size acini. The acini contain prostatic secretions in the lumen. The acini are lined with simple columnar epithelium ((**A**), 200×). Fine stroma and a continuous basal layer are also noted ((**B**), 400×) (control group). (**C**), (**D**): Prostate sections from the untreated BPH animals show irregular acinar shape with papillary projection into the lumen, and foci of piling-up hyperplastic nodules are evident. ((**C**), 200× and (**D**), 400×). The epithelium is highly cylindrical, multilayered, and shows irregularly aligned round/ovoid nuclei. (**E**), (**F**): Prostate sections from BPH animals treated with aescin show marked reduction in hyperplasia and hypertrophy ((**E**), 100× and (**F**), 200×).

**Figure 3:**
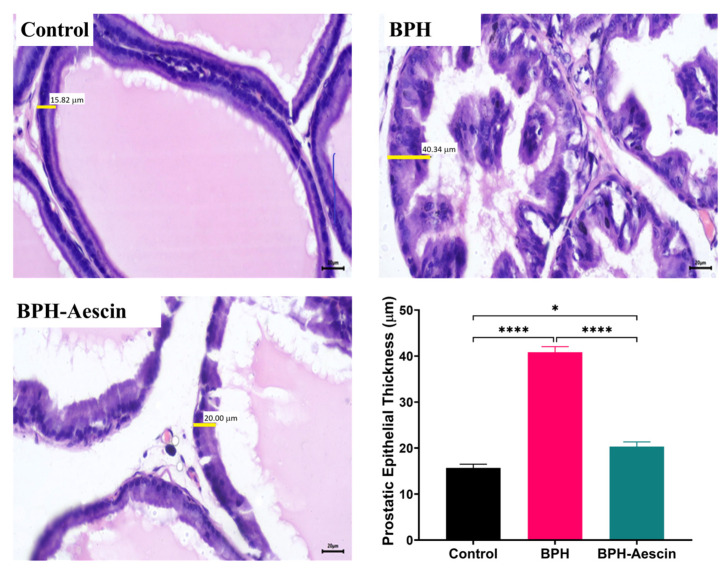
Aescin abrogates the testosterone-induced epithelial thickening in the prostate. Sections of rat prostate stained with Hematoxylin–Eosin from the control, BPH, and BPH rats treated with aescin (10 mg/kg, p.o.) for 4 weeks are shown (400×). The representative photomicrographs show the thickness (yellow bars) of prostate glandular epithelium among the different studied groups. The bar graph in the lower-right corner shows the analysis of prostatic epithelial tissue thickness measurements. Data (Mean ± SEM, *n* = 5) were analyzed by one-way ANOVA followed by Tukey’s multiple comparisons test. * and **** denote significant differences between the selected study groups at *p* values < 0.05 and 0.0001, respectively.

To test whether proliferative changes in the prostate epithelium accompanied the increase in prostate weight and prostate weight index, we used light microscopy to examine the tissue sections (Figure 2 and Figure 3). Histological examination of prostatic sections from the control group showed no morphological changes in acinar size and shape or in the lining epithelium of the acini of prostatic tissue. In the control group, the lining epithelium comprises cuboidal epithelial cells of regular size (Figure 2A,B). The prostatic tissue of the untreated BPH animal group developed a disturbance in the shape and the size of prostatic acini. The acini showed epithelial prostatic hyperplasia with papillary projections in the lining epithelium of the acini and widening of the lumen (Figure 2C,D). These features were consistent with BPH pathology. Contrary to the untreated BPH rat prostates, the aescin-treated BPH rats showed only mild epithelial hyperplasia (Figure 2E,F). Analysis of the prostatic epithelial layer thickness data showed maximized prostatic epithelial thickness in the untreated BPH rats. This effect was completely mitigated by concomitant aescin treatment, Figure 3.

### 2.3. Effect of Aescin Treatment on Serum Testosterone and Luteinizing Hormone

**Figure 4:**
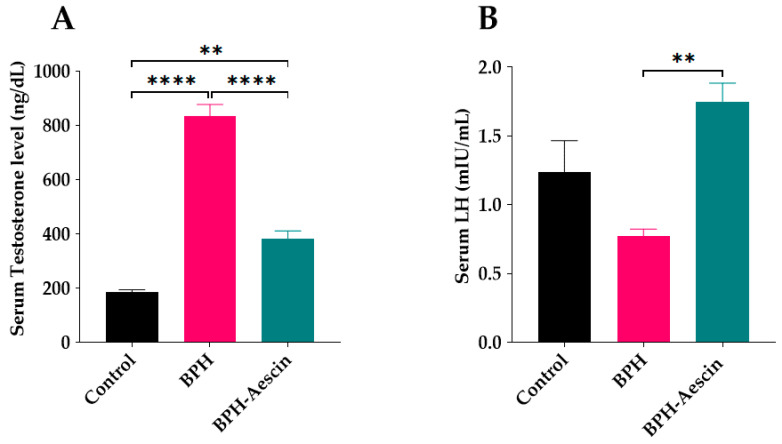
Aescin antagonizes testosterone-induced serum hormonal abnormalities. Serum samples were collected from control, BPH, and BPH–aescin groups, and were used for determination of: (**A**) serum testosterone and (**B**) luteinizing hormone (LH). The concentrations of testosterone or LH were determined by electrochemiluminescence immunoassay “ECLIA” kits as described in Section 4 Materials and Methods. Rats were subjected to a 4-week treatment protocol of sesame oil (control), testosterone (3 mg/kg, s.c.) dissolved in sesame oil (BPH), or testosterone (3 mg/kg, s.c.) dissolved in sesame oil + aescin (10 mg/kg, p.o.) (BPH–aescin). Data (mean ± SEM, n = 5) were analyzed by one-way ANOVA followed by Tukey’s multiple comparisons test. ** and **** denote significant differences between the selected study groups at *p* values < 0.01 and 0.0001, respectively.

Rats in the untreated BPH group showed a significant elevation of serum testosterone (834.2 ± 43.56 ng/dL) in comparison with the control rats (186.1 ± 8.137 ng/dL), as shown in Figure 4A. On the other hand, aescin treatment significantly ameliorated such elevation of serum testosterone (383.4 ± 28.03 ng/dL) in comparison with the untreated BPH group. However, serum testosterone was still significantly higher than in the control rats. Induction of BPH by testosterone for 4 weeks insignificantly reduced serum luteinizing hormone (LH) concentrations compared with the control rats (0.77 ± 0.05 vs. 1.24 ± 0.23 mIU/L, respectively, Figure 4B), while rats treated with aescin showed significantly increased serum levels of LH compared with the untreated BPH rats (1.75 ± 0.13 vs. 0.77 ± 0.05 mIU/mL, respectively).

### 2.4. Effect of Aescin Treatment on Prostatic Expression of IL1-β, TNF-α, and TGF-β1

**Figure 5:**
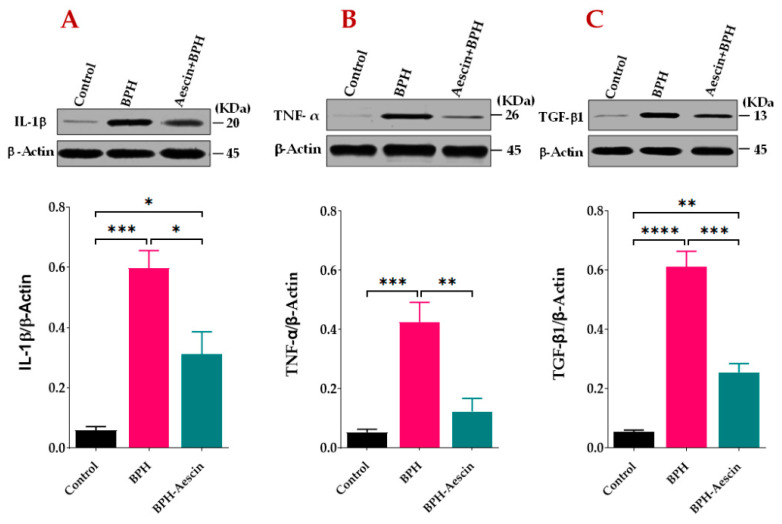
Aescin attenuates the testosterone-induced expression of IL-1β, TNF-α, and TGF-β1 in the prostate. Prostate tissue samples harvested from control, BPH, and BPH–aescin groups were homogenized in lysis buffer containing protease inhibitor cocktail and further processed for SDS-PAGE followed by Western blotting on nitrocellulose membranes as described in Section 4 Materials and Methods. The membranes were probed with mouse monoclonal antibodies against IL-1β (**A**), TNF-α (**B**), or TGF-β1 (**C**), and the protein expression was normalized by β-actin. The immunoblots (upper panel) are representations of 4 independent experiments. Data (Mean ± SEM, *n* = 4) were analyzed by one-way ANOVA followed by Tukey’s multiple comparisons test (lower panel). *, **, ***, and **** denote significant differences between the selected study groups at *p* values < 0.05, 0.01, 0.001, and 0.0001, respectively.

We tested the anti-inflammatory effects of aescin on the progression of testosterone-induced BPH in rats. As the data in Figure 5A,B show, the prostates from untreated BPH rats showed significantly (*p* < 0.001) increased protein levels of IL-1β and TNF-α by approximately 10- and 8-folds, respectively. Concomitant administration of aescin and testosterone during the induction of BPH significantly attenuated the testosterone-induced elevated expression of these inflammatory mediators. Although the prostates of aescin-treated BPH rats showed normalized levels of TNF-α (*p* = 0.55), these tissues showed significantly higher than normal levels of IL-1β (5-fold vs. controls, *p* < 0.05). The expression of TGF-β1 was determined as a marker of tissue proliferation. The data in Figure 5C show that untreated BPH rats displayed an 11.37-fold increase in TGF-β1 in comparison with the control group (*p* < 0.0001). Comparable with its effect on prostate IL-1β levels, aescin treatment significantly inhibited such an increase by 58.30% (*p* < 0.001). However, these tissues displayed higher than normal levels of TGF-β1 (a 4.73-fold increase compared with the control group, *p* < 0.01).

### 2.5. Effect of Aescin on the Expressions of PCNA and COX-2 in the Prostate of BPH Rats

**Figure 6:**
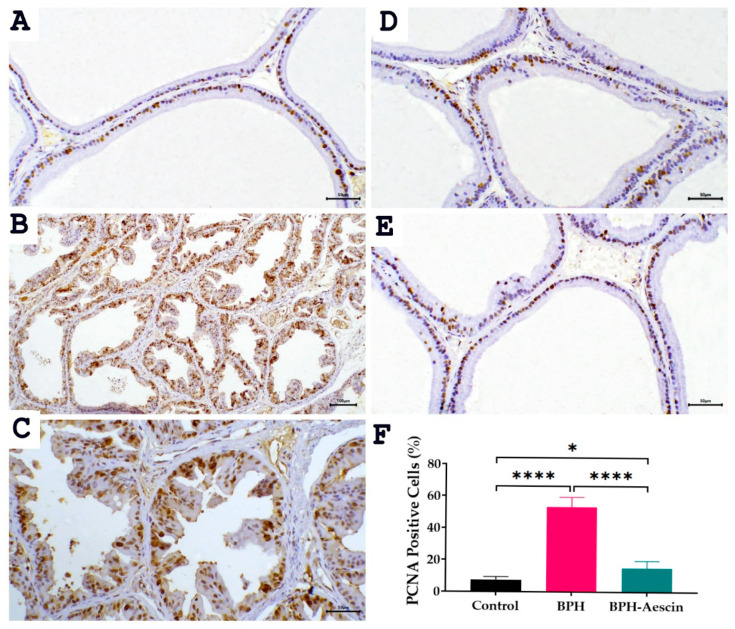
Effect of aescin treatment on the expression of prostate PCNA. The representative photomicrographs show immunohistochemical staining of proliferating cell nuclear antigen (PCNA) in prostate tissues among various studied groups. (**A**): Normal prostate tissue showing focal nuclear expression of PCNA staining among the epithelial cells ((**A**), 200×). The prostate tissue of the untreated BPH group shows a marked increase in PCNA immunostaining ((**B**), 100× and (**C**), 200×). Prostatic tissue from animal groups treated with aescin ((**D**) and (**E**) 200×) show less frequent immunostaining of PCNA. Quantitative analysis of PCNA expression in rat prostates among different studied groups is also shown (**F**). Significant elevation in the frequency of PCNA positive cells was observed within prostatic acini in the untreated BPH group compared with the normal control group, which was significantly decreased by aescin treatment. Data in the bar chart (mean ± SEM of 5 tissues) were analyzed by one-way ANOVA followed by Tukey’s multiple comparisons test. * and **** denote significant differences between the selected study groups at *p* values < 0.05 and 0.0001, respectively.

The proliferating cell nuclear antigen (PCNA) is an established marker of tissue proliferation. We measured the level of PCNA protein expression by immunohistochemistry in prostate tissues from the control, BPH, and BPH–aescin groups. Data in Figure 6 illustrate the prostatic expression of PCNA (photomicrographs) and its quantitation (bar chart) in the different animal groups. The data show that PCNA staining is confined to the nuclei. Although the nuclear staining varied in intensity, all identifiable staining was considered positive. In the normal control group, we observed a few PCNA-positive cells estimated at 7.5 ± 0.76%, distributed in a single or focal pattern. Compared with controls, the expression of PCNA protein was significantly elevated in the prostate tissues of the BPH group, in which the PCNA positive cells were increased in prostatic acini to 53.33 ± 2.47% (*p* < 0.001). On the other hand, the percentage of PCNA positive cells was reduced in the acini of the aescin-treated BPH group to 15.0 ± 1.83%.

**Figure 7:**
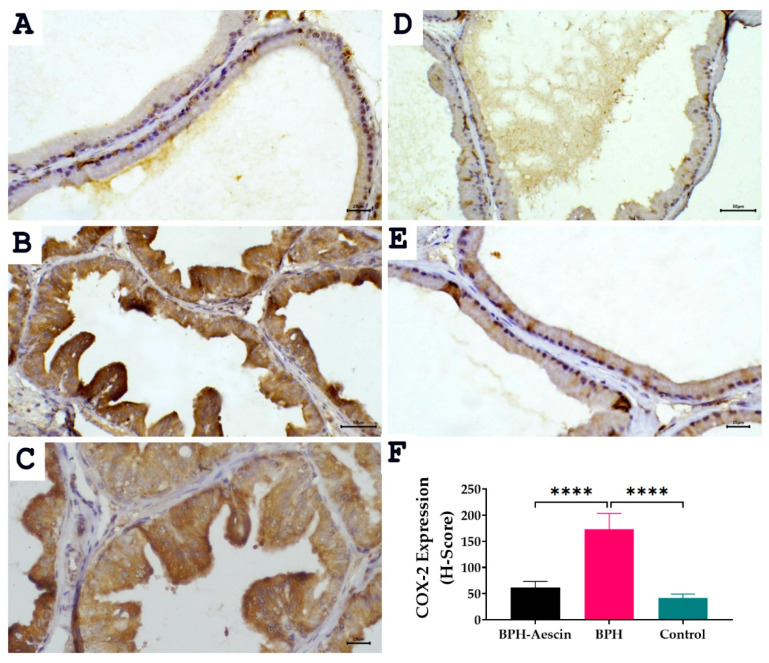
Effect of aescin treatment on the expression of prostate COX-2. The representative photomicrographs show the immunohistochemical staining of COX-2 in the prostate tissues among various studied groups. (**A**): Normal prostate tissue showing faint COX-2 staining (400×). Prostate tissue of the BPH group shows diffuse marked increase in cytoplasmic COX-2 immuno-expression ((**B**), 200× and (**C**), 400×). The prostatic tissue from animal groups treated with aescin shows a marked decrease in COX-2 immuno-expression ((**D**), 200× and (**E**), 400×). The bar graph (**F**) shows semi-quantitative analysis of COX-2 expression in the rat prostates among the different studied groups expressed as H-score values as described in Section 4 Materials and Methods. A significant elevation in COX-2 expression was observed within prostatic acini in the BPH group compared with either the normal control or the aescin-treated BPH groups. Data in the bar chart (mean ± SEM of 5 tissues) were analyzed by one-way ANOVA followed by Tukey’s multiple comparisons test. **** denote significant differences between the selected groups at *p* < 0.0001.

The expression of the inducible cyclooxygenase isoform COX-2 was determined by immunohistochemistry in prostate tissue sections from the three studied groups. Data in Figure 7 revealed positive COX-2 expression shown as brown cytoplasmic staining in the prostate epithelial cells. Akin to PCNA expression, COX-2 expression increased significantly in the testosterone-induced untreated BPH group compared with the control group (173.3 ± 12.22 vs. 41.67 ± 3.07, respectively, *p* < 0.0001). Whereas COX-2 levels in the aescin-treated BPH group were significantly lower than in the untreated BPH group (61.67 ± 4.77 vs. 173.3 ± 12.22, respectively, *p* < 0.0001).

### 2.6. Effect of Aescin Treatment on Serum ALT Activity and Creatinine Level

**Figure 8:**
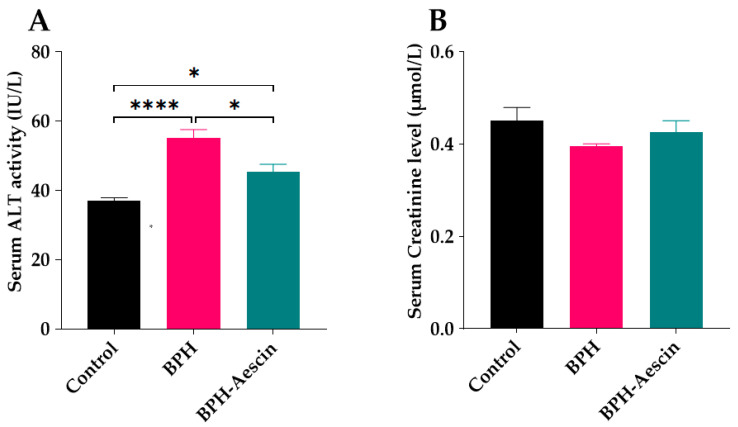
Effect of aescin on serum of ALT and creatinine levels in BPH rats. Serum samples were collected from control, BPH, and BPH–aescin groups, and were used for determination of the activity ALT (**A**) as a hepatic function marker, and creatinine level (**B**) as a surrogate of renal function. Colorimetric assay kits were used in these tests. Rats were subjected to a 4-week treatment protocol of sesame oil (control), testosterone (3 mg/kg, s.c.) dissolved in sesame oil (BPH), or testosterone (3 mg/kg, s.c.) dissolved in sesame oil + aescin (10 mg/kg, p.o.) (BPH–aescin). Data (mean ± SEM, *n* = 5) were analyzed by one-way ANOVA followed by Tukey’s multiple comparisons test. * and **** denote significant differences between the selected study groups at *p* values < 0.05 and 0.0001, respectively.

We tested the effect of continued administration of testosterone alone or with aescin on liver and kidney function by determination of the serum activity of ALT and the concentration of creatinine, respectively. As shown in Figure 8A, daily administration of testosterone (3 mg/kg, s.c.) for four weeks to induce BPH in the rats moderately elevated the serum ALT activity compared with the control rats (55.0 ± 2.55 vs. 37.0 ± 0.84 IU/L, respectively, *p* < 0.0001). BPH rats treated with aescin showed significantly lower ALT activity in comparison with the untreated BPH rats. However, the ALT activity was still somewhat higher, although slightly, than that observed in the control animals (45.0 ± 2.09 vs. 37.0 ± 0.84 IU/L, respectively, *p* < 0.05). Figure 8B shows that neither testosterone alone (BPH) nor its combination with aescin (BPH–aescin) significantly altered the serum creatinine level compared with control rats.

## 3. Discussion

Benign prostatic hyperplasia (BPH) is the most common disease condition in aging males [2,9]. Prostate inflammation and irregular growth of prostate cells are characteristic features of BPH that contribute to the development of lower urinary tract symptoms [2]. The major contributing factors in the pathogenesis of BPH are inflammation, cellular proliferation, and oxidative stress [12,19,20]. We investigated the protective effects of the natural saponin aescin against the development of BPH induced by daily testosterone injection in rats. Aescin mitigated the testosterone-induced epithelial hyperplastic changes, attenuated prostate tissue inflammation, and preserved the normal histology of the prostate to levels comparable with those in the control normal rats. To the best of our knowledge, these results are the first to show that aescin protects against the development of BPH in experimental rats.

In the current study, induction of BPH by daily testosterone administration resulted in hyperplasia of the prostate as illustrated by the increased prostate weight and the ratio of prostate weight-to-body weight in the untreated BPH rats. Microscopical examination of tissue sections confirmed these results by showing increased epithelial thickness, irregular morphology, and hyperplastic nodules. Aescin treatment of BPH rats has normalized these parameters, which shows its protective effects against testosterone-induced BPH. In addition, aescin-treated rats showed lower serum testosterone, albeit still higher than normal values. This effect might contribute to the protective action of aescin against BPH development. The testosterone-induced proliferative effects in the prostate were positively correlated with serum testosterone levels (Supplementary Materials), which is in line with the findings of recent work by others [37]. Previous studies showed that protection against testosterone-induced BPH by a combination of natural extracts decreased the serum levels of testosterone and its more potent derivative dihydrotestosterone (DHT) [38]. Others showed a treatment-mediated decrease in DHT [39]. In female rats, aescin decreased serum testosterone levels in a model of polycystic ovary syndrome [40]. These results substantiate the aescin-induced reduction in serum testosterone we observed in the current study. In tissues, 5α-reductase converts testosterone to DHT. Either testosterone or DHT binds to and activates androgen receptors (AR) [9,41]. Besides its nuclear translocation and activation of AR-dependent genes, the testosterone-bound AR activates the cytoplasmic non-receptor tyrosine kinase Src via protein–protein interaction. Src kinase can activate the epidermal growth factor receptor and its downstream signaling pathways to suppress apoptosis and induce cell proliferation and survival [42].

Although the pathophysiology of BPH is yet to be fully characterized, activation of inflammatory signals is key to the pathogenesis of BPH [9,19]. The extent of hyperplasia and inflammatory changes largely determines the severity of LUTS in patients with BPH [2,17,18]. Tissue inflammation in the prostate results from the interplay between hormonal abnormalities, vascular alterations, and deranged metabolism in conditions such as diabetes, obesity, and metabolic syndrome [17]. A careful review of the literature shows that infiltration of prostatic tissue by immune cells magnifies tissue proliferation and increases the expression of inflammatory cytokines, such as TNF-α and IL-1β [7,8].

In experimental animal models, exogenous testosterone activates several inflammatory pathways, including the increased expression of proinflammatory cytokines, infiltration of leukocytes, and subsequent activation of inflammatory genes [11,20,43]. This mechanism turns on an inflammatory vicious cycle by increasing the expression of proinflammatory cytokines, such as TNF-α and IL-1β, and other inflammatory genes, e.g., COX-2 [3]. In line with these effects, induction of BPH by daily injection of testosterone in the current study elevated the prostate expression of TNF-α, IL-1β, and COX-2 in BPH rats. On the contrary, concurrent treatment of the rats with aescin alleviated the testosterone-induced prostate inflammation, tissue proliferation, and histopathological changes. Nonetheless, our results show that aescin treatment did not completely block tissue inflammation in the testosterone-treated rats as evident by higher-than-normal levels of IL-1β and positive, although at low levels, COX-2 staining.

Inflammatory signaling promotes further inflammation by overexpression of COX-2. Evidence showed that COX-generated arachidonate-derived pro-inflammatory prostaglandins further exaggerate local inflammation and abnormal growth of the prostate [22,43,44]. In addition, increased COX-2 inhibits apoptotic death of damaged prostatic tissue and supports the proliferation of prostatic tissue in BPH [43,44]. Another effect of COX-2 overexpression is its alteration of redox balance, which can activate redox-sensitive cytokine-dependent downstream targets [44] to further intensify the inflammatory response. This signaling paradigm agrees with our data: BPH rats showed high levels of COX-2, TNF-α, and IL-1β, while aescin treatment could attenuate the expression of COX-2 in the prostate through inhibition of the inflammatory-cytokine/NFκB/COX-2 pathway. This is in coherence with the results of previous studies, which support our notion [3,28,45,46]. Besides, aescin might induce anti-inflammatory and antioxidant effects by induction of heme oxygenase (HO)-1 expression [28,47], like other natural phytochemicals [44,46]. However, whether this later antioxidant effect of aescin contributed to its protection against the development of BPH is yet to be tested.

The imbalance between prostate cell apoptosis and proliferation is a major contributing factor in the progression of hyperplasia of prostatic tissue [9,19,25]. Previous studies showed that prostatic inflammation increases the expression of TGF-β, which directly controls the development of BPH and its related symptoms [19,26] as well as prognosis [24,48]. Activation of TGF-β signaling sustains the proliferative over the apoptotic activity in prostatic cells and induces epithelial-to-mesenchymal transition (EMT) and tissue remodeling [25,49]. Study of testosterone-induced BPH in animal models showed that testosterone disrupts the balance between proliferative and antiproliferative factors via increased TGF-β1 signaling [20,50]. Moreover, the expression of TGF-β and its activity correlates with androgen receptor signaling during prostate inflammation [22], and modulation of this pathway proved protective against androgen-mediated BPH progression [20,51].

In this study, we observed increased prostatic levels of TGF-β1 in BPH rats, which was attenuated by concurrent aescin treatment. This effect can be directly linked to the increased PCNA nuclear staining observed in the BPH rats, which is also mitigated by aescin. Previous studies provide strong evidence on the connection between TGF-β activation, increased nuclear PCNA expression, and tissue proliferation [16,37]. Increased PCNA leads to accumulation of mesenchymal-like cells derived from prostatic epithelial tissue [49]. The current results show that, in comparison with the normal rats, the BPH–aescin rats displayed modest elevation in TGF-β1, which was paralleled with increased PCNA expression. Furthermore, the results of the correlation analysis between the study parameters in the current work show a strong positive correlation between the percentage of PCNA positive cells and tissue levels of IL-1β1, TNF-α, or TGF-β1, in increasing order (Supplementary Materials). Thus, our results provide further evidence that both inflammatory cytokine-mediated and TGF-β-mediated pathways contribute to epithelial tissue proliferation in BPH. Aescin showed antiproliferative effects with a noticeable reduction in prostate weight through mitigating the expression of TGF-β1, and therefore, repressing the testosterone-induced PCNA elevation in BPH rat prostates. In cancer cells, previous studies documented the ability of aescin to activate proapoptotic signaling (e.g., caspase-3) and arrest the cell cycle [33,52]. The findings by others [53] showing that aescin induces the binding and activation of the cyclin-dependent kinase inhibitor p21 to PCNA resulting in cell-cycle arrest at the G1-S phase might help explain its antiproliferative activity. By lowering (or destabilizing) nuclear PCNA, aescin could restore the homeostasis between cell death and proliferation in the prostate.

The anti-inflammatory effects of aescin were established in several inflammatory conditions [45,54,55]. Its triterpene moiety confers structural similarity to cortisol; this explains its glucocorticoid-like and anti-inflammatory effects [27]. Via its glucocorticoid-like effect, aescin might interfere with signaling by inflammatory transcription factors through genomic and non-genomic mechanisms [30]. The possible genomic mechanisms involve its binding to the glucocorticoid receptors, nuclear translocation, and binding to the glucocorticoid response element, followed by transrepression of inflammatory cytokines such as TNF-α and IL1β. The non-genomic glucocorticoid-like actions of aescin might be diverse; they include the direct interaction between the ligand-bound glucocorticoid receptors and modulators of other signaling pathways such as Src-kinase as well as other protein-kinase pathways, and direct interaction with inflammatory transcription factors in the nucleus [27,56]. A recent study reported that intracerebral hemorrhage disrupts the blood–brain barrier (BBB) function via activation of IL-1β/RhoA/ROCK/NFκB signaling, which results from increased degradation of the inhibitor of κB (IκB) [55]. However, aescin treatment suppressed the expression of IL-1β, maintained the IL-1β/RhoA/ROCK/NFκB pathway at basal activity, and restored the BBB function, an effect that was reversed by exogenous IL-1β administration [55]. Since activation of RhoA/ROCK can activate NFκB signaling by increasing the degradation of IκB [57], thus inhibiting the IL-1β/RhoA/ROCK cascade, aescin can also decrease the degradation IκB. These results confirm the IL-1β-lowering effect of aescin observed in our study. Previous studies have documented increased expression of glucocorticoid receptors upon aescin treatment [54,58,59], which augments the endogenous glucocorticoid-mediated anti-inflammatory effects.

Because prolonged androgen administration may negatively impact liver and kidney function, [60,61] we tested the liver and kidney function of rats by the end of the study period by simple laboratory means. Only mild negative changes were observed in the untreated BPH rats regarding liver function, while the kidney function (serum creatinine) remained unchanged. Since the testosterone-induced changes in liver and kidney function were only subtle or absent, respectively, and because we only measured serum ALT and creatinine, the results of the current study do not provide evidence on liver or kidney protection by aescin. Nonetheless, the current results show that aescin treatment did not have adverse effects on either renal or hepatic function.

Fortunately, previous experimental evidence showed that aescin lacks immunosuppression [62] or interference with physiological wound healing usually encountered with glucocorticoids [63]. Clinical evidence showing the high tolerability of aescin in thyroid cancer patients supports these previous findings [64]. Thus, because of its potent anti-inflammatory effects and the lack of glucocorticoid-like adverse effects, aescin is a promising anti-inflammatory agent. Moreover, aescin may have an advantage over finasteride, a 5α-reductase inhibitor heavily used to treat BPH [4,5,6]. Chronic inhibition of 5α-reductase shifts the fate of testosterone metabolism to the formation of estradiol by the action of the aromatase enzyme, which causes sexual dysfunction [5,11].

Although our results show for the first time that aescin protects against the development BPH and prevents prostate tissue remodeling, the current study has some limitations. One important limitation is that we did not study the role of oxidative stress in the current work. However, oxidative stress is known to contribute to the progression of BPH [1], and antioxidants protect against its development [43,44,50]. Moreover, the antioxidant potential of aescin has been verified by various studies [47,56,58,59], while contradictory reports by others showed that it can promote reactive oxygen species generation [34]. Thus, whether modulation of redox balance contributes to the observed aescin-mediated protection against BPH remains open for future investigations. Another limitation of the current study is the limited number of samples per group (*n* = 5) although a 10-animal per group design was followed; half of the tissue samples were used in histopathology and immunohistochemistry studies.

## 4. Materials and Methods

### 4.1. Animals

Male Wistar rats (12 weeks old), weighing 180–200 g were purchased from the experimental animal facility of the Nahda University in Beni-Suef (NUB), Egypt. Animals were housed in a 12/12 h light/dark air-conditioned atmosphere (25 °C ±1) in PVC cages and allowed free access to tap water and standard animal chow during the study period. Rats were acclimatized for 10 days before starting the experiment. All experimental protocols were approved by “The Commission on the Ethics of Scientific Research”, Faculty of Pharmacy, Minia University (number ES12/2020).

### 4.2. Drugs and Chemicals

The aescin used in the study was amorphous aescin powder (CAS: 6805-41-0), isolated from the seeds of *Aesculus hippocastanum L.* (Horse chestnut seed), with known therapeutic activity: >95% triterpene glycosides (residual solvents: <5% of water, ethanol, and traces of methanol), and was a kind gift from the Chemical Industries Development Company (CID), Giza, Egypt. Thiopental sodium (batch number 1806554) and testosterone oenanthate (batch number 20601001) were obtained from EIPICO (10^th^ of Ramadan City, Egypt) and CID, respectively. Electrochemiluminescence immunoassay kits for testosterone (Elecsys Testosterone II; cat. No. 05200067) and luteinizing hormone (Elecsys LH; cat no. 11732234122) were purchased from Roche Diagnostics, Mannheim, Germany.

Mouse monoclonal antibodies against TNF-α, IL-1β, and TGF-β1 were purchased from Santa-Cruz Biotechnology (Heidelberg, Germany), while β-actin mouse monoclonal antibodies were from R&D SYSTEMS, Minneapolis, MN, USA. Alkaline phosphatase-tagged anti-mouse antibody, 5-bromo-4-chloro-3-indolyphosphate (BCIP), and nitro-blue tetrazolium (NBT) were purchased from Sigma-Aldrich, St. Louis, MO, USA. All other chemicals were of analytical grade.

### 4.3. Experimental Design

Rats were randomly allocated into three groups (10 rats/group). The control group received only the vehicle (sesame oil, 1 mL/kg), while the BPH group received testosterone oenanthate (3 mg/kg/day, s.c.) dissolved in sesame oil for BPH induction according to previously described methods [20,43]. The aescin-treated BPH rats received aescin (10 mg/kg/day, p.o.) + testosterone oenanthate (3 mg/kg/day, s.c.) dissolved in sesame oil. The treatment protocol continued for four weeks. The dose of aescin was selected based on preliminary work results in our lab (unpublished data) and pervious literature [65].

### 4.4. Sample Collection and Preparation

At the end of the study, animals were fasted overnight and weighed. Blood was collected via cardiac puncture from thiopental-anesthetized rats (50 mg/kg, i.p.) prior to euthanasia by exsanguination. Coagulated blood was centrifuged at 10,000 rpm for 10 min to collect serum samples. The serum activity of alanine aminotransferase (ALT) and the serum levels of creatinine were determined colorimetrically by commercially available kits as markers of liver and kidney function, respectively.

The prostate gland was carefully dissected, weighed, washed in cold PBS, and blotted dry on a filter paper. A portion of the prostate was flash-frozen in liquid nitrogen and kept at −20 °C for further analyses. Another portion of the prostate tissue was fixed overnight in 10% formalin/saline solution and used for the histopathological and immunohistochemical assessments. The prostate weight index (mg/g) was calculated by dividing the weight of the prostate tissue (mg) by the animal body weight (g), as previously described [50].

### 4.5. Determination of Serum Levels of Testosterone and Luteinizing Hormone (LH)

Concentrations of testosterone or LH were measured in serum samples by electrochemiluminescence immunoassay “ECLIA” kits following the manufacturer’s instructions. The assay principle depends on the binding of the free hormone (testosterone or LH) in the sample to biotinylated monoclonal hormone-specific antibodies followed by adding a ruthenium-bound derivative of the hormone (supplied in the kit reagents) to the mixture to compete for the antibody binding sites. The labeled complex, after fixation on streptavidin-coated microparticles, is magnetically attracted to the surface of an electrode, where voltage application induces chemiluminescence that is captured by a photomultiplier.

### 4.6. Western Blotting Analysis of TNF-α, IL-1β, and TGF-β1

Estimation of prostate tissue protein expression of TNF-α, IL-1β, and TGF-β1 was carried out by Western blotting after SDS-PAGE electrophoresis as previously described [66]. Briefly, the total protein concentrations were determined in the supernatants of 10% (w/v) prostate tissue homogenates prepared in phosphate-buffered saline containing protease inhibitors (Nacalai Tesque, Inc., Kyoto, Japan). Samples were then mixed with 2× sample buffer and subjected to denaturing at 95 °C for 3 min. A volume of denatured sample equivalent to 30 μg total protein was subjected to SDS-PAGE. Protein bands were transferred to a nitrocellulose membrane using a semi-dry blotter (Bio-Rad, California, USA). The blot was subsequently blocked with 5% skim milk in tris-buffered saline with 0.1% Tween 20 (TBST) for 1 h at room temperature. Then, the blot was probed overnight at 4 °C with mouse monoclonal antibodies against TNF-α, IL-1β, TGF-β1 (Santa-Cruz Biotechnology, Heidelberg, Germany), or β-actin (R&D SYSTEMS, Minneapolis, MN, USA). Subsequently, the membranes were incubated with alkaline phosphatase-tagged anti-mouse antibodies for 1 h at room temperature. Blots were finally washed and visualized using the 5-bromo-4-chloro-3-indolyl phosphate (BCIP)/nitro-blue tetrazolium (NBT) colorimetric detection method. We analyzed protein bands on the blots using the ImageJ software packages (version 1.5k) developed at the National Institute of Health, Bethesda, Maryland, USA [67].

### 4.7. Histopathological Assessment

The formalin-fixed tissue samples were paraffin-embedded and sectioned at a thickness of 5 μm. Dewaxed and rehydrated prostate sections were mounted on slides and stained with hematoxylin–eosin (H&E) for microscopical examination by light microscopy using Olympus CX23 (Olympus, Tokyo, Japan) fitted with LED illumination and a trinocular tube. Blinded pathological assessment of the histopathological changes was carried out for each group. We measured the thickness of the prostatic epithelial layer in the five most central acini at 400× magnification.

### 4.8. Immunohistochemical detection of PCNA and COX-2

A streptavidin-biotin immunoperoxidase complex procedure was applied in the immunohistochemical detection of PCNA and COX-2. Sections on adhesive slides measuring 5-μm-thick from representative formalin-fixed, paraffin-embedded blocks were deparaffinized and dehydrated as per the standard procedures [68]. Endogenous peroxidase activity was blocked by incubation by treatment with 0.3% hydrogen peroxide in methanol for 30 min. Antigen retrieval of PCNA and COX-2 was done by microwave treatment (15 min) in sodium citrate buffer (pH 6). Tissue sections were then incubated overnight at 4 °C with rabbit anti-PCNA (Santa Cruz, 1:30 dilution) for 30 min at room temperature and rabbit anti-COX-2 (Abcam, 1:100 dilution). The sections were washed and incubated with biotinylated anti-rabbit secondary antibodies for 30 min (room temperature). The reaction was then visualized with an avidin–biotin complex immunoperoxidase system using 3,3′ diaminobenzidine (DAB) as a chromogen. Sections were then counterstained with hematoxylin, dehydrated, cleared, and mounted with distyrene, plasticizer, and xylene (DPX).

For PCNA, nuclear staining was considered positive. The labeling index of PCNA was calculated as the number of positive cells that showed nuclear immunoreactivity in ten representative microscopic fields, and the percentage of positive cells was then calculated relative to the total number of nuclei.

For COX-2, diffuse brown cytoplasmic staining in the prostate epithelial cells was considered positive. Semi-quantitative assessment of the intensity (scored 0–3), and estimation of the percentage of cells positive at each intensity in 10 high-powered fields was determined. The two measurements were multiplied and summed to give an H-score varying from 0 to 300. An H score cut-off value of 50 was considered positive, while a score ≥ 100 was considered strong positivity, according to previous reports [69].

### 4.9. Statistical Analysis

Results were expressed as mean ± standard error of the mean (SEM) and were analyzed for statistically significant differences using one-way analysis of variance (ANOVA) followed by the Tukey–Kramer post-analysis test using GraphPad Prism^®^ 7 software (San Diego California, USA). Two-tailed *p* values were calculated and the differences between groups were significant at *p* < 0.05.

## 5. Conclusions

In conclusion, the results of the current study showed, for the first time, the potential of aescin as a novel agent in the prophylaxis and management of experimental BPH. Aescin-induced suppression of TNF-α/IL-1β/COX-2 and TGF-β1/PCNA are possible mechanisms of its anti-inflammatory and antiproliferative effects, respectively. These mechanisms, at least in part, underlie aescin-mediated protection against the progression of BPH. Because of its better safety over other anti-inflammatories and conventional BPH medications, aescin can be a promising agent for treating BPH. The results of this work, besides the availability of approved systemic aescin preparations, warrant further clinical studies to test the feasibility of repurposing aescin for BPH management.

## Data Availability

Data is contained within the article and Supplementary Materials.

## Supplementary Materials

The following are available online at https://www.mdpi.com/article/10.3390/ph15020130/s1, Figure S1: Correlation matrix between the study parameters.
