# Supplementary material for: Aescin Protects against Experimental Benign Prostatic Hyperplasia and Preserves Prostate Histomorphology in Rats via Suppression of Inflammatory Cytokines and COX-2
Source: Pharmaceuticals (Basel). 2022 Jan 22;15(2):130. doi: 10.3390/ph15020130 (PMC8880638; doi:10.3390/ph15020130)
Supplement: Supplementary file 1 [file pharmaceuticals-15-00130-s001.zip › pharmaceuticals-1486258-supplementary.pdf]

Data Analysis:

Since all data sets passed the D'Agostino & Pearson test for normality or lognormality, the correlation between the measured parameters was determined by calculation of the parametric Pearson correlation coefficient, r. The correlation is weak when r <|0.3|, moderate when |0.3| ≤ r ≤ |0.7|, and strong when r > |0.7|.

Correlation analysis of the measured parameters

Data in Figure 8 illustrate the relationship between the results of the current study in the form of a correlation matrix showing the Pearson's correlation coefficient between the measured parameters. The data show that serum testosterone level has a strong positive correlation with the prostate proliferation (prostate weight, prostate weight index, epithelial thickness, PCNA, and TGF-β) and inflammation (IL-1β, TNF-α, and COX-2) parameters, and negative moderate correlation with serum LH. Of note, the tissue levels of either TNF-α and TGF-β show strong positive correlation among themselves and between either of them and the prostate expression of either PCNA or COX-2. Serum LH is moderately negatively correlated with all the studied parameters.

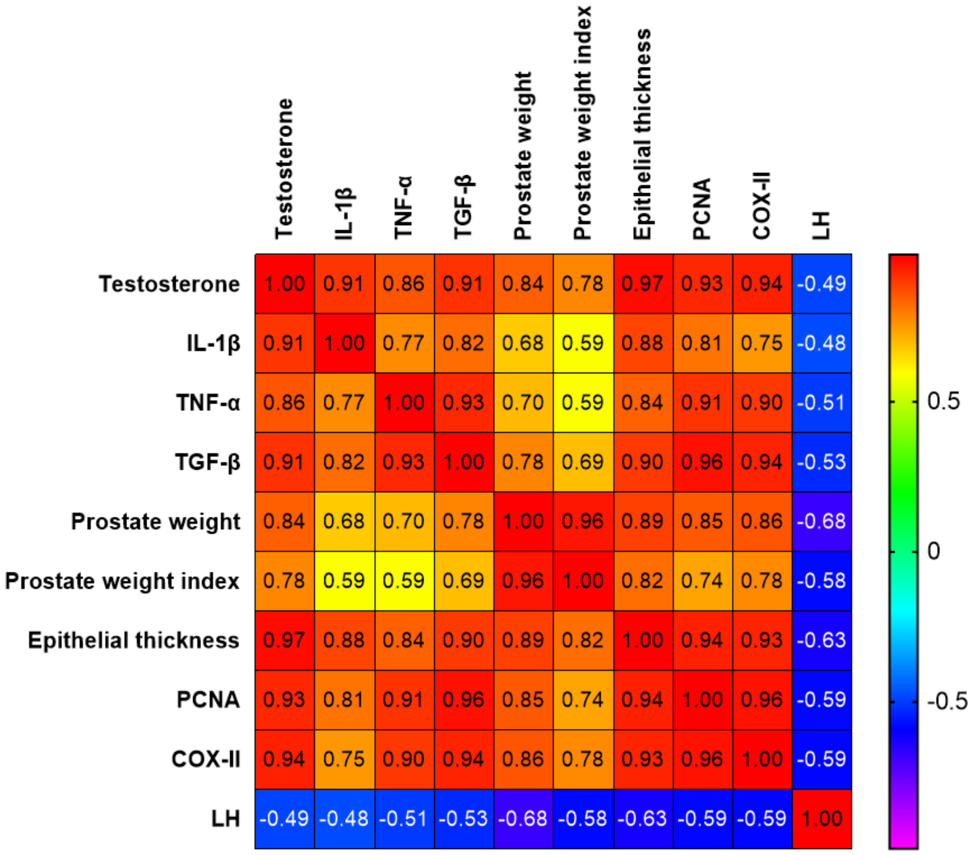

Figure S1. Correlation matrix between the study parameters. Data from different experiments were analyzed for correlation using the Pearson correlation coefficient (r). Positive values indicate a positive correlation while negative values indicate a negative one. When r is in the range of |0.3|–|0.7|, this indicates a moderate correlation, and when r >|0.7|, this means a strong correlation. The graph is rainbow-color-coded so that the red and violet colors indicate r values of +1 and −1, respectively, see the scale bar to the right. IL-1β: interleukin-1β; TNF-α: tumor necrosis factor α; TGFβ: transforming growth factor β; Prostate weight index was calculated as prostate weight (mg) divided by the body weight (g); PCNA: proliferating cell nuclear antigen; COX-2: cyclooxygenase-2; LH: luteinizing hormone.
